# Skeletonisation contributing to a reduction of sternal wound complications: a retrospective study in OPCAB patients

**DOI:** 10.1186/s13019-019-0985-9

**Published:** 2019-09-09

**Authors:** Jef Van den Eynde, Astrid Heeren, Delphine Szecel, Bart Meuris, Steven Jacobs, Peter Verbrugghe, Wouter Oosterlinck

**Affiliations:** Department of Cardiovascular Diseases, Research Unit of Cardiac Surgery, University Hospitals Leuven, KU Leuven, Herestraat 49, 3000 Leuven, Belgium

**Keywords:** CABG, Diabetes mellitus, Infections, Internal mammary artery, OPCAB, Skeletonisation, Sternal wound complications, Sternal wound infections

## Abstract

**Background:**

Sternal wound complications (SWC) are a rare but potentially life-threatening complication after coronary artery bypass grafting (CABG) surgery. Especially the use of bilateral IMA (BIMA) grafts as opposed to single IMA (SIMA) grafts is associated with an increased risk of SWC. Skeletonised harvesting has been proposed to reduce this risk. The purpose of this study was to retrospectively investigate the effect of skeletonisation on SWC after off-pump coronary artery bypass grafting (OPCAB) in a centre with a high volume of off-pump procedures and high frequencies of BIMA.

**Methods:**

From January 2010 to November 2016, 1900 consecutive patients underwent OPCAB surgery at the University Hospitals of Leuven. The first group (*n* = 1487) received non-skeletonised IMA grafts, whereas the second group (*n* = 413) received skeletonised grafts. Optimal wound management was pursued in all patients. A new four-grade classification for SWC was developed. Incidence and grade of SWC as well as overall survival were assessed.

**Results:**

Analysis of diabetic patients showed a lower incidence of SWC in the skeletonised (12/141, 8.5%) compared to the non-skeletonised group (82/414, 19.8%) [odds ratio 0.46, 95% confidence interval (0.23;0.88), *p* = 0.019] as well as a lower grade [0.45 (0.24;0.871), *p* = 0.018]. There was no significant effect on overall survival [0.67 (0.19;2.32), *p* = 0.529]. Subanalysis of this population revealed that the observed effects were most prominent in patients receiving BIMA grafts, with 6/56 (10.7%) SWC in the skeletonised and 62/252 (24.6%) in the non-skeletonised group [0.37 (0.15;0.90), *p* = 0.028 for incidence], as well as a lower grade [0.36 (0.15;0.88), *p* = 0.025]. These advantages were not significant in diabetic patients receiving SIMA grafts nor in the full study population.

**Conclusions:**

This study, using a more sensitive classification of SWC, shows in a large group of patients that, in combination with optimized wound management, the skeletonisation technique is associated with a clear reduction in the incidence and grade of SWC in diabetic patients receiving BIMA grafts. This encourages the extension of BIMA use in OPCAB to this risk population.

**Electronic supplementary material:**

The online version of this article (10.1186/s13019-019-0985-9) contains supplementary material, which is available to authorized users.

## Background

The internal mammary artery (IMA) has become increasingly popular as a conduit for coronary artery bypass grafting (CABG) [1]. Many studies have demonstrated its advantages of long-term patency, improved resistance to the development of arteriosclerosis, and better overall survival [2–5]. Bilateral IMA grafting (BIMA) as compared to single IMA grafting (SIMA) might even further improve cardiac protection and reduce the need for repeat revascularisation [6–10]. A major drawback of BIMA however is the increased risk of sternal wound complications (SWC) associated with the conventional non-skeletonised technique of IMA harvesting [11, 12]. SWC is an infrequent complication, but can be potentially life-threatening and lead to prolonged hospitalisation as well as increased costs due to repeated surgical intervention [13, 14]. This is especially problematic in patients with already existing risk factors for SWC, such as diabetic patients [12, 15]. At the same time, these patients could probably derive the greatest benefit from BIMA.

Skeletonisation of IMA grafts was suggested as a technique that could reduce this risk for SWC [16, 17]. The collateral sternal blood supply and the internal mammary veins are preserved in this technique, thereby inducing less devascularisation of the sternum [18, 19]. Skeletonisation could therefore provide a way to reduce the incidence of SWC while maintaining the benefit of using both IMAs. The existing literature is however still inconclusive. As the skeletonisation technique is also more difficult to perform and could involve a risk of damage to the endothelium and surrounding tissues, a decisive answer is required.

Several smaller studies have already demonstrated a lower proportion of SWC after on-pump CABG if skeletonisation was used [17, 20–22]. However, only a limited amount of studies investigated the use of skeletonised grafts in off-pump coronary artery bypass grafting (OPCAB) [23, 24]. Although an active discussion about the relative benefits of both techniques is still ongoing, some studies suggested that OPCAB is associated with less morbidity postoperatively, including less infections [25]. The combination of OPCAB with skeletonised grafting might provide even better outcomes. Data from the Arterial Revascularization Trial (ART) showed significantly higher rates of early SWC after BIMA, and lower rates of SWC when skeletonisation was performed, regardless of whether single or bilateral grafts were used [26]. Interestingly, a post hoc analysis of the trial data showed that patients receiving BIMA grafts tended to have lower rates of SWC if they underwent OPCAB compared to on-pump CABG [27].

The purpose of this study was therefore to retrospectively investigate the effect of skeletonisation on SWC after OPCAB in a centre with a large volume of off-pump procedures and high frequencies of BIMA. We aimed to study these effects both in the entire patient population and more specifically in the high risk group of patients with diabetes mellitus.

## Methods

### Population and study design

This study conforms to the ethical guidelines of the 1975 Declaration of Helsinki as reflected in a priori approval by the local Ethical Committee of the University Hospitals of Leuven. The data from 1900 consecutive patients who underwent OPCAB surgery at the University Hospitals of Leuven between January 2010 and November 2016, were studied. Between January 2010 and December 2014, conventional non-skeletonised technique was used to harvest the IMA in all patients (*n* = 1487). From January 2015 to November 2016, all patients (*n* = 413) received a skeletonised IMA graft. Every patient undergoing OPCAB surgery was considered, including both elective and urgent (non-elective) surgeries as well as both SIMA and BIMA. Patients were followed up based on information available in their electronic medical records, as well as hospitalisations and outpatient surgical and cardiology consultations. Minimal period of follow-up for all patients was until discharge or death. Groups were made by the harvesting technique used during surgery: skeletonised versus non-skeletonised.

Demographic characteristics and perioperative variables were considered and compared between both groups. Demographics included age, gender and body mass index (BMI). Comorbidities such as diabetes, renal insufficiency (eGFR < 30 ml/min), organ transplantation, COPD with need of bronchial dilatation treatment, and use of oral corticosteroids were analysed. Diabetes was further classified: 1 for diet-controlled diabetes, 2 for diabetes controlled with oral medicines, and 3 for diabetes with need of insulin treatment. Perioperative information contained the harvesting method, the use of SIMA or BIMA, and whether the surgery was an emergency. The major postoperative outcomes were the incidence and grade of SWC. Another outcome was the overall survival.

Because commonly used classifications such as the STS classification of deep sternal wound infections only include severe types of infection, a new classification is introduced in this study for a more comprehensive definition of wound complications. This classification, as given in Table 1, is made up of four severity grades. Grade 1 and 2 are mild and superficial sternal wound problems or infections with minimal impact on patient recovery, while grade 3 and 4 are severe and deep complications that need subsequent surgical interventions. The majority of the SWC were diagnosed at the time of hospitalisation.

**Table 1:** Grading of sternal wound complications

Grade 1: Minor	Superficial wound problem: local redness or minimal drainage Conservative approach, spontaneous healing
Grade 2: Superficial	Wound infection: positive culture Antibiotic treatment
Grade 3: Moderate	Deep wound infection: soft tissue dehiscence without bone extension Need for drainage, debridement, or VAC
Grade 4: Severe	Mediastinitis or mechanical sternal dehiscence with bone extension Refixation of the sternum or omentoplasty

*VAC* vacuum assisted closure

### Surgical procedure

The operations were performed by a team of 7 surgeons. Optimal wound management was pursued in all patients. Preoperative infection prophylaxis has widely remained the same in the period covered, only the incision drapes have become iodinated over the years. Patients had a preoperative chlorhexidine bath and the surgical site was clipped. Antibiotic prophylaxis was given by intravenous administration of 3 g of cefazolin before incision and 2 g every 3 h intraoperatively. Tight glycemic control was attained pre-, per- and postoperatively. A median sternotomy was performed in all patients and all surgeries were off-pump coronary artery bypasses (OPCAB). Bone wax was used according to needs. The sternum was closed using 8 to 10 single wires and subsequently the fascia, subcutis and skin were closed each separately using running sutures. In limited circumstances, such as extremely skinny or obese patients or patients with severe COPD, the figure-of-eight technique was used for closure of the sternum instead of sigle wires.

When the skeletonised technique was used, the IMA was isolated from the thoracic wall using meticulous dissection, leaving the adjacent vein, fat tissue, endothoracic fascia, parietal pleura and intercostal muscle undisturbed. Dissection was performed using electrocautery, and arterial side branches were divided using hemoclips and microscissors. In the non-skeletonised technique, the IMA was dissected along with the surrounding tissues using electrocautery. All IMA grafts were mobilised from the first rib to the bifurcation of the IMA into the superior epigastric and musculophrenic arteries.

### Statistical analyses

Continuous variables were checked for normality and the difference between groups was tested with the t-test or Mann-Whitney U test accordingly. Categorical variables are expressed as frequency and proportion, and differences were assessed with the Chi-square test. Logistic regression analysis was used to analyse the group effect on incidence of SWC, a proportional odds model was used to analyse SWC grade, and a Cox proportional hazards model was used for the assessment of overall survival. Correction for group differences was performed by using multivariable models, including these patient characteristics in the analysis model on which groups were shown to differ. The analyses of SWC incidence and grade were performed (a) including all grades of SWC and (b) excluding grade 1 SWC by means of sensitivity analysis. Overall survival was also estimated with Kaplan-Meier curves. All tests were two-sided and a *p*-value less than 0.05 was deemed statistically significant. All analyses have been performed using SAS software (version 9.4; SAS Institute, Cary, NC).

## Results

### Entire study population

**Fig. 1:**
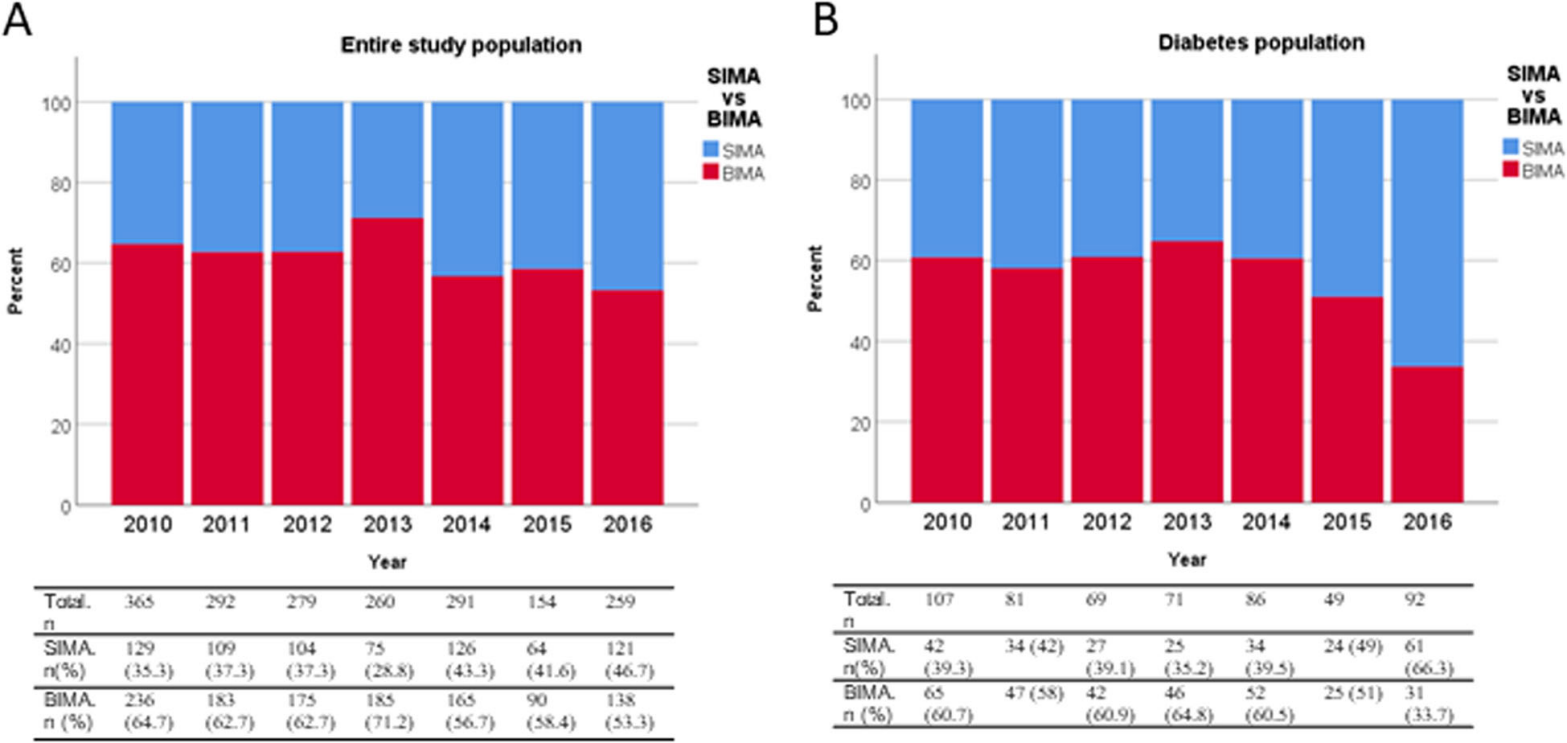
Histograms showing the proportion of BIMA use for each year. **a** in the entire study population. **b** in diabetic patients

**Fig. 2:**
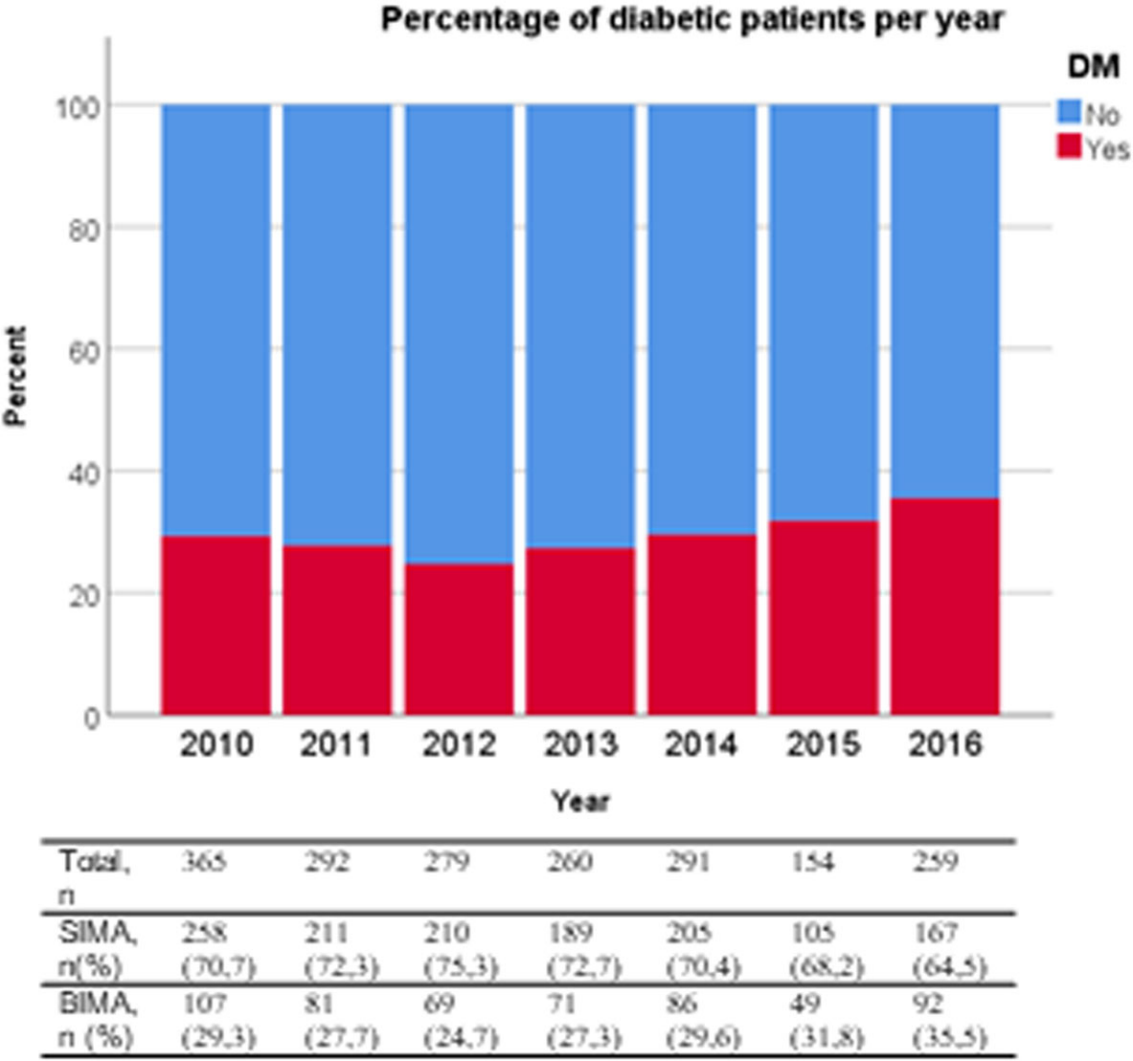
Histogram showing the proportion of diabetic patients for each year

**Fig. 3:**
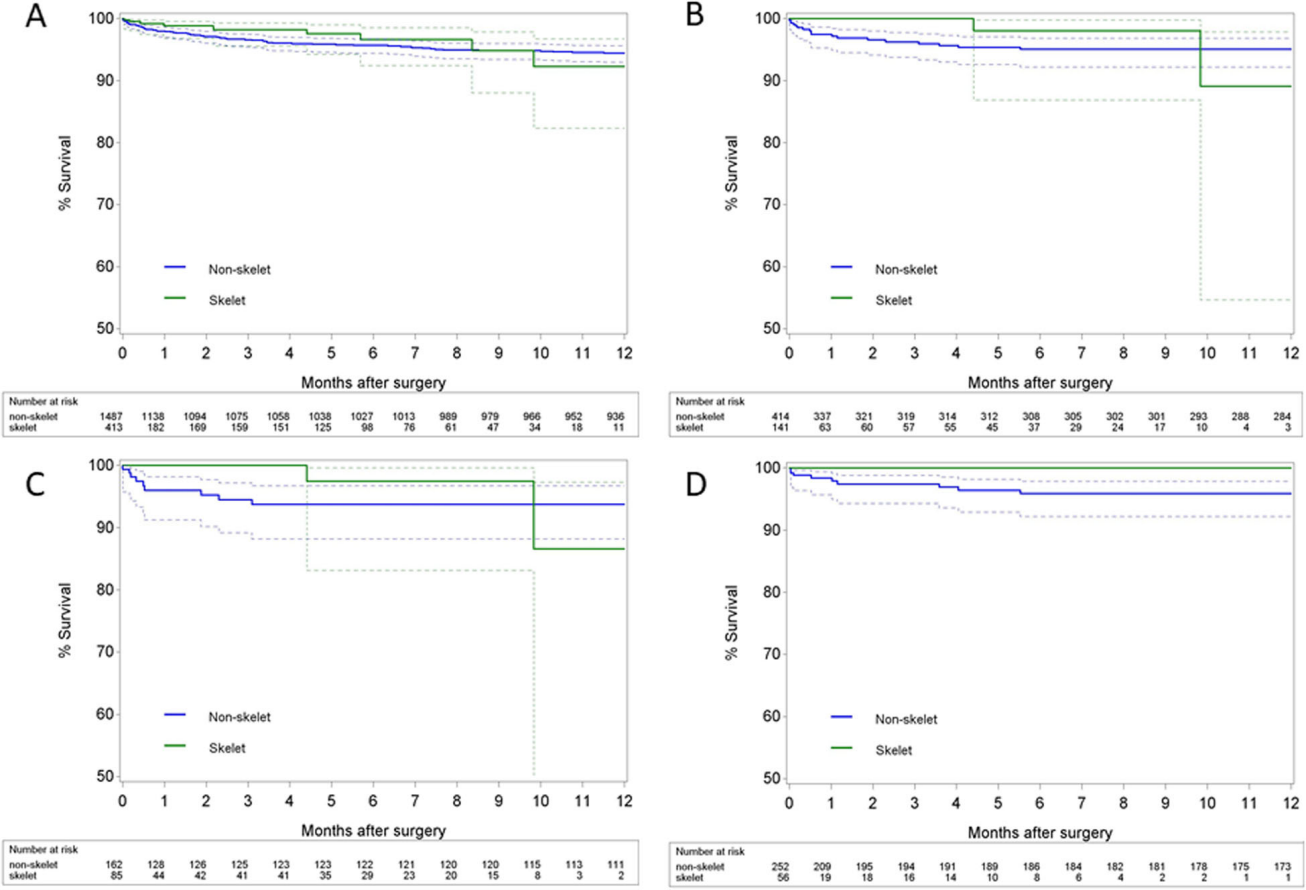
Kaplan-Meier curves showing overall survival. **a** in the entire study population. **b** in the diabetes subgroup. **c** in diabetes patients who received SIMA grafts. **d** in diabetes patients who received BIMA grafts

Demographic characteristics of the entire study population are given in Table 2. Patients in both groups were similar for most variables except for a greater prevalence of diabetes mellitus in the skeletonised group (34.14% vs 27.84% for skeletonised and non-skeletonised grafts, respectively, *p* = 0.015) and a lower proportion of BIMA grafts (55.21% vs 63.48%, *p* = 0.002). Figure 1a gives the proportion of BIMA use per year, and the proportion of diabetic patients per year is represented in Fig. 2. Median follow-up time was 326 days (interquartile range (IQR) 21–1261 days). As shown in Table 3, patients receiving skeletonised IMA grafts had no significantly lower incidence [9.44% vs 12.78%, odds ratio 0.74, 95% confidence interval (0.51;1.07), *p* = 0.114] or grade [0.73 (0.50;1.05), *p* = 0.091] of SWC than those receiving non-skeletonised grafts. After exclusion of grade 1 SWC in the analyses, a reduction was seen which was however not significant after correction for demographics. There was also no apparent beneficial effect in overall survival [0.92 (0.48;1.75), *p* = 0.791] (Fig. 3a). The complete multivariable models are given in the Additional file 1.

**Table 2:** Demographic characteristics and perioperative variables of the entire study population

Variable	Non-skeletonized (*n* = 1487)	Skeletonized (*n* = 413)	*P*
Age	67.8 ± 9.79	68.0 ± 9.14	0.956
Male gender	1201 (80.77)	333 (80.63)	0.950
BMI	26.9 ± 4.28	27.2 ± 4.49	0.899
BMI group			
< 25	393 (28.23)	119 (29.10)	0.752
25–29	663 (47.63)	188 (45.97)	
30–34	274 (19.68)	79 (19.32)	
> 34	62 (4.45)	23 (5.62)	
Urgent surgery	703 (47.28)	210 (50.85)	0.199
Diabetes mellitus			
0	1073 (72.16)	272 (65.86)	0.015
1	40 (2.69)	7 (1.69)	
2	241 (16.21)	92 (22.28)	
3	133 (8.94)	42 (10.17)	
Oral corticosteroids	126 (8.47)	26 (6.30)	0.149
Transplantation	11 (3.09)	6 (1.45)	0.173
Renal insufficiency	46 (3.09)	18 (4.36)	0.208
COPD	90 (6.05)	27 (6.54)	0.717
BIMA	944 (63.48)	228 (55.21)	0.002

*BMI* Body mass index, *COPD* chronic obstructive pulmonary disease, *BIMA* bilateral internal mammary artery bypass grafting

Values are presented as mean ± SD or n (%)

*P* < 0.05 was considered significant

**Table 3:** Outcomes: sternal wound complication incidence and grade and overall survival in the entire study population

Variable	Non-skeletonized (n = 1487)	Skeletonized (n = 413)	Odds ratio (95% CI)	*P*
SWC incidence	190 (12.78)	39 (9.44)	0.71 (0.50;1.02)	0.067
			0.74 (0.51;1.07)^a^	0.114^a^
SWC incidence (grade 1 excluded)	116 (7.80)	20 (4.84)	0.60 (0.37;0.98)	0.041
			0.635 (0.387;1.042)	0.072^a^
SWC grade			0.70 (0.49;1.01)	0.054
			0.73 (0.50;1.05)^a^	0.091^a^
SWC grade (grade 1 excluded)			0.59 (0.36;0.97)	0.036
			0.62 (0.38;1.02)	0.062^a^
1	74 (4.98)	19 (4.60)		
2	27 (1.82)	10 (2.42)		
3	63 (4.24)	7 (1.69)		
4	26 (1.75)	3 (0.73)		
Overall survival (%, 95 CI)			1.00 (0.52;1.91)	1
			0.92 (0.48;1.75)^a^	0.791^a^
1 month	97.99 (97.08;98.62)	98.81 (96.82;99.56)		
6 months	95.67 (94.36;96.68)	96.61 (92.39;98.50)		
12 months	94.42 (92.94;95.60)	92.25 (82.32;96.71)		

*SWC* sternal wound complication, *CI* confidence interval

^a^Corrected for diabetes mellitus and BIMA

Values are presented as mean ± SD or n (%) if not otherwise specified

*P* < 0.05 was considered significant

### Diabetic patients

The interaction term between skeletonisation and diabetes mellitus within the entire study population was statistically significant for both SWC incidence (*p* = 0.039) and grade (*p* = 0.042), suggesting that the effect of skeletonisation was dependent on the presence or absence of diabetes mellitus. The analysis was therefore performed for the subpopulation of diabetic patients. Between January 2010 and November 2016, 555 diabetic patients underwent OPCAB. The non-skeletonised technique was used in 414 of them, while 141 diabetic patients received a skeletonised IMA graft. As shown in Table 4, demographic characteristics and comorbidities were similar between the two groups except for a higher proportion of BIMA grafts in the skeletonised group of diabetic patients (39.72% vs 60.87%, respectively, *p* < 0.001). The proportion of BIMA use in diabetic patients per year is represented in Fig. 1b. Median follow-up time was 336 days (IQR 30–1302 days). Table 5 reveals that diabetic patients who underwent OPCAB with a skeletonised IMA graft had a significantly lower incidence of SWC [8.51% vs 19.81%, 0.46 (0.24;0.88), *p* = 0.019] and a lower grade of SWC [0.45 (0.24;0.87), *p* = 0.018]. After exclusion of grade 1 SWC, the corrected difference was not significant anymore however. There was however no significant effect on overall survival [0.67 (0.19;2.32), *p* = 0.529] (Fig. 3b).

**Table 4:** Demographic characteristics and perioperative variables of the diabetes subgroup

Variable	Non-skeletonized (*n* = 414)	Skeletonized (*n* = 141)	*P*
Age	67.4 ± 9.16	67.8 ± 8.55	0.626
Male gender	325 (78.50)	110 (78.01)	0.903
BMI	28.0 ± 4.84	28.2 ± 4.79	0.908
BMI group			
< 25	80 (20.57)	34/140 (24.29)	0.638
25–29	180 (46.27)	63/140 (45.00)	
30–34	99 (25.45)	30/140 (21.43)	
> 34	30 (7.71)	13/140 (9.29)	
Urgent surgery	198 (47.83)	73 (51.77)	0.418
Diabetes mellitus			
1	40 (9.66)	7 (4.96)	0.150
2	241 (58.21)	92 (65.25)	
3	133 (32.13)	42 (29.79)	
Oral corticosteroids	34 (8.21)	13 (9.22)	0.711
Transplantation	5 (1.21)	4 (2.84)	0.186
Renal insufficiency	20 (4.83)	11 (7.80)	0.185
COPD	24 (5.80)	14 (9.93)	0.093
BIMA	252 (60.87)	56 (39.72)	< 0.001

*BMI* Body mass index, *COPD* chronic obstructive pulmonary disease, *BITA* bilateral internal mammary artery bypass grafting

Values are presented as mean ± SD or n (%)

*P* < 0.05 was considered significant

**Table 5:** Outcomes: sternal wound complication incidence and grade and overall survival in the diabetes subgroup

Variable	Non-skeletonized (*n* = 414)	Skeletonized (*n* = 141)	Odds ratio (95% CI)	*P*
SWC incidence	82 (19.81)	12 (8.51)	0.38 (0.20;0.71)	0.003
			0.46 (0.24;0.88)^a^	0.019^a^
SWC incidence (grade 1 excluded)	53 (12.80)	8 (5.67)	0.41 (0.19;0.88)	0.023
			0.47 (0.22;1.03)^a^	0.058^a^
SWC grade			0.38 (0.20;0.71)	0.003
			0.45 (0.24;0.87)^a^	0.018^a^
SWC grade (grade 1 excluded)			0.41 (0.19;0.88)	0.022
			0.47 (0.21;1.02)^a^	0.055^a^
1	29 (7.00)	4 (2.84)		
2	11 (2.66)	3 (2.13)		
3	32 (7.73)	4 (2.84)		
4	10 (2.42)	1 (0.71)		
Overall survival (%, 95 CI)			0.74 (0.21;2.54)	0.630
			0.67 (0.19;2.32)^a^	0.529^a^
1 month	97.42 (95.26;98.61)	100.00		
6 months	95.02 (92.20;96.84)	98.04 (86.88;99.72)		
12 months	95.02 (92.20;96.84)	89.13 (54.65;97.83)		

*SWC* sternal wound complication, *CI* confidence interval

^a^Corrected for BIMA

Values are presented as mean ± SD or n (%) if not otherwise specified

*P* < 0.05 was considered significant

The interaction term between skeletonisation and BIMA vs SIMA use was not significant for SWC incidence (*p* = 0.326) and grade (*p* = 0.284). However, a sub-analysis was performed for diabetic patients receiving SIMA grafts and those receiving BIMA grafts separately, because of clinical relevance. Demographics of these subdivisions were similar (Tables 6 and 7). Median follow-up times were 281 days (IQR 21–1201.5 days) and 519 days (IQR 30–1369.5 days), respectively. Diabetic patients undergoing OPCAB with SIMA grafts had no significantly lower incidence [7.06% vs 12.35%, 0.54 (0.21;1.40), *p* = 0.204] or grade [0.55 (0.21;1.42), *p* = 0.217] of SWC than those receiving non-skeletonised grafts (Table 8). However, patients receiving BIMA grafts had a considerable advantage of skeletonisation, with a lower incidence [10.71% vs 24.60%, 0.37 (0.15;0.90), *p* = 0.028] and grade [0.36 (0.15;0.88), *p* = 0.025] of SWC (Table 9). These results were similar after exclusion of grade 1 SWC in the analyses. In both SIMA and BIMA, overall survival between skeletonised and non-skeletonised was similar (Fig. 3c and d).

**Table 6:** Demographic characteristics and perioperative variables of the diabetes subgroup receiving SIMA grafts

Variable	Non-skeletonized (*n* = 162)	Skeletonized (*n* = 85)	*P*
Age	70.5 ± 9.04	70.5 ± 7.80	0.672
Male gender	119 (73.46)	63 (74.12)	0.911
BMI	27.4 ± 4.79	28.0 ± 4.94	0.684
BMI group			
< 25	37 (25.34)	22 (25.88)	0.759
25–29	61 (41.78)	39 (45.88)	
30–34	38 (26.03)	17 (20.00)	
> 34	10 (6.85)	7 (8.24)	
Urgent surgery	85 (52.47)	49 (57.65)	0.438
Diabetes mellitus			0.331
1	15 (9.26)	4 (4.71)	
2	85 (52.47)	51 (60.00)	
3	62 (38.27)	30 (35.29)	
Oral corticosteroids	14 (8.64)	9 (10.59)	0.617
Transplantation	2 (1.23)	3 (3.53)	0.224
Renal insufficiency	11 (6.79)	10 (11.76)	0.183
COPD	12 (7.41)	10 (11.76)	0.253

*BMI* Body mass index, *COPD* chronic obstructive pulmonary disease

Values are presented as mean ± SD or n (%)

*P* < 0.05 was considered significant

**Table 7:** Demographic characteristics and perioperative variables of the diabetes subgroup receiving BIMA grafts

Variable	Non-skeletonized (*n* = 252)	Skeletonized (*n* = 56)	*P*
Age	65.5 ± 8.72	63.8 ± 8.12	0.276
Male gender	206 (81.75)	47 (83.93)	0.700
BMI	28.3 ± 4.85	28.5 ± 4.59	0.863
BMI group			
< 25	43 (17.70)	12 (21.82)	0.780
25–29	119 (48.97)	24 (43.64)	
30–34	61 (25.10)	13 (23.64)	
> 34	20 (8.23)	6 (10.91)	
Urgent surgery	113 (44.84)	24 (42.86)	0.787
Diabetes mellitus			
1	25 (9.92)	3 (5.36)	0.254
2	156 (61.90)	41 (73.21)	
3	71 (28.17)	12 (21.43)	
Oral corticosteroids	20 (7.94)	4 (7.14)	0.841
Transplantation	3 (1.19)	1 (1.79)	0.722
Renal insufficiency	9 (3.57)	1 (1.79)	0.495
COPD	12 (4.76)	4 (7.14)	0.468

*BMI* Body mass index, *COPD* chronic obstructive pulmonary disease

Values are presented as mean ± SD or n (%)

*P* < 0.05 was considered significant

**Table 8:** Outcomes: sternal wound complication incidence and grade and overall survival in the diabetes subgroup receiving SIMA grafts

Variable	Non-skeletonized (*n* = 162)	Skeletonized (*n* = 85)	Odds ratio (95% CI)	*P*
SWC incidence	20 (12.35)	6 (7.06)	0.54 (0.21;1.40)	0.204
SWC incidence (grade 1 excluded)	12 (7.41)	5 (5.88)	0.78 (0.27;2.30)	0.653
SWC grade			0.55 (0.21;1.42)	0.217
SWC grade (grade 1 excluded)			0.78 (0.27;2.29)	0.649
1	8 (4.94)	1 (1.18)		
2	3 (1.85)	2 (2.35)		
3	8 (4.94)	2 (2.35)		
4	1 (0.62)	1 (1.18)		
Overall survival (%, 95 CI)			0.57 (0.12;2.65)	0.469
1 month	95.98 (91.24;98.18)	100.00		
6 months	93.71 (88.23;96.69)	97.44 (83.16;99.63)		
12 months	93.71 (88.23;96.69)	86.61 (47.47;97.26)		

*SWC* sternal wound complication, *CI* confidence interval

Values are presented as mean ± SD or n (%) if not otherwise specified

*P* < 0.05 was considered significant

**Table 9:** Outcomes: sternal wound complication incidence and grade and overall survival in the diabetes subgroup receiving BIMA grafts

Variable	Non-skeletonized (n = 252)	Skeletonized (n = 56)	Odds ratio (95% CI)	*P*
SWC incidence	62 (24.60)	6 (10.71)	0.37 (0.15;0.90)	0.028
SWC incidence (grade 1 excluded)	41 (16.27)	3 (5.36)	0.29 (0.09;0.98)	0.046
SWC grade			0.36 (0.146;0.88)	0.025
SWC grade (grade 1 excluded)			0.29 (0.09;0.97)	0.044
1	21 (8.33)	3 (5.36)		
2	8 (3.17)	1 (1.79)		
3	24 (9.52)	2 (3.57)		
4	9 (3.57)	0 (0.00)		
Overall survival (%, 95 CI)			0.93 (0.11;7.63)	0.946
1 month	98.35 (95.65;99.38)	100.00		
6 months	95.87 (92.19;97.84)	100.00		
12 months	95.87 (92.19;97.84)	100.00		

*SWC* sternal wound complication, *CI* confidence interval

Values are presented as mean ± SD or n (%) if not otherwise specified

*P* < 0.05 was considered significant

## Discussion

### Main findings

Despite suggested superiority regarding graft patency and long-term survival [6–10], BIMA grafting for CABG has remained largely underutilised [28]. This has been primarily due to perceived challenges such as longer surgical times and a higher risk of SWC [11, 12]. As severe SWC is associated with longer and more expensive hospital stay and higher in-hospital mortality [14], the use of the IMA is often limited to one graft. The present study, which defined SWC widely including both superficial and deep infections, found that SWC is a complication that still affects 12.78% of patients undergoing OPCAB with conventional non-skeletonised IMA grafting. This finding is comparable to that reported by De Paulis et al. [17] and Peterson et al. [20]. Mediastinitis was seen in 1.75% of the patients. Given the impact of this complication, the use of BIMA should indeed be carefully considered.

The conventional way for IMA harvesting is the non-skeletonised technique, which might lead to significant sternal devascularisation. Since sternal ischemia is one of the major factors in the pathophysiology of SWC, skeletonisation was proposed as an alternative technique that would better preserve sternal vascularisation [16, 17]. Cohen et al. [19] used single photon emission computer tomography to show that sternal blood flow was not significantly reduced after skeletonised IMA grafting, as opposed to non-skeletonised grafting. Kamiya et al. [29] could similarly demonstrate that there was less deterioration in sternal oxygen saturation and microcirculation with the skeletonisation technique. Additionally, Sá et al. [30] showed skeletonised IMA to be non-inferior in comparison to non-skeletonised IMA regarding patency, despite more manipulation of the graft.

Several smaller studies have already pointed towards superiority of skeletonised over non-skeletonised harvesting in CABG [17, 20–22]. However, proportions of BIMA use in these studies were low. Additionally, the benefits of skeletonised harvesting have only poorly been described in OPCAB [29, 31]. In our hospital, the use of BIMA in combination with OPCAB is a common practice, with over 95% of all coronary revascularisations being performed off-pump. When compared to the 4–12% BIMA use reported by the meta-analysis of Weiss et al. [32], the frequencies in our study (63.48 and 55.21% for skeletonised and non-skeletonised, respectively) were high. Whereas other studies showed results that contained either no OPCAB or pooled data from both on-pump and off-pump procedures, our study included OPCAB procedures only. Our patients can thus be considered as an ideal study population to investigate SWC as well as the risks and gains of skeletonisation in OPCAB. With 1900 patients, this was a large study and was thus provided with enough power to detect clinical advantages.

The main finding of our study is that skeletonisation was associated with a lower incidence (10.7% vs 24.6%, OR 0.37) and grade (OR 0.36) of SWC in diabetic patients after OPCAB with BIMA grafts. These results remained significant after exclusion of grade 1 SWC from the analyses. In accordance with the ART trial, SWC incidence was higher after BIMA, but skeletonisation of these grafts allowed to reduce SWC incidence in diabetic patients, from 24.60 to 10.71% in and achieve results that are more comparable to those after SIMA grafting (12.35% without skeletonisation and 7.06% with skeletonisation) [26]. This is especially relevant, as CABG is still a golden standard treatment in diabetic patients because outcomes of percutaneous transluminal coronary angioplasty are still unfavorable in this population [33]. In addition, the population undergoing CABG increasingly contains larger numbers of diabetic patients, as was shown in this study (Fig. 2). We therefore suggest that skeletonisation might help in realising the full potential of BIMA in diabetic patients and that the use of BIMA might be safely expanded to this risk group without considerably increasing the risk for SWC.

In our study, the interaction term between skeletonisation and BIMA vs SIMA use was not statically significant. Even though we noticed a significant effect of skeletonisation on SWC incidence and grade in patients receiving BIMA grafts, we can therefore not conclude that skeletonisation has absolutely no effect when SIMA grafts are used. Most likely, skeletonisation has an effect in SIMA, but the effect size is smaller, such has been demonstrated by the ART trial and is suggested by our results (OR 0.54 for SWC incidence after SIMA vs 0.37 after BIMA) [26]. As such, our study might have failed to detect these effects due to relative lack of statistical power. However, the subdivision of our analyses into the two groups was clinically meaningful, as the the question whether skeletonisation is effective in preventing SWC is especially pertinent in the setting of diabetes patients receiving BIMA grafts.

The choice for BIMA use might further be motivated by other studies showing advantages in long-term outcomes such as all-cause survival, hospital mortality rates, cerebrovascular accidents and need for revascularisation, all of which could outweigh the short-term increase in SWC [6–10, 34]. For instance, the meta-analysis of 27 observational studies totaling 79,063 patients by Weis et al. showed a long-term survival benefit of BIMA compared to SIMA (hazard radio 0.78, CI 0,72-0,84) [32]. Nonetheless, we have to note that these benefits have not been confirmed by the recent ART trial, which concluded that both had equal outcomes [26]. The benefit of BIMA on long-term survival might be age-related however, as Toumpoulis et al. demonstrated that patients 60–69 year had better 5-year survival rates after BIMA compared to SIMA, whereas the opposite was seen in patients older than 79 [35]. Additionally, other studies have already demonstrated that skeletonisation allows to reduce SWC rates after on-pump BIMA [17, 20, 22]. Furthermore, Matsa et al. showed that operative mortality and incidence of deep sternal wound infection were similar between diabetic and nondiabetic patients [22]. We argue therefore that BIMA should be considered as a reasonable option in patients that could benefit from it.

Despite demonstrated benefits, revascularisation by OPCAB using BIMA grafts is generally little-used. Instead, SIMA and a venous or alternative arterial graft are often used to achieve complete revascularisation. This might be due to concerns about SWC associated with BIMA. Our and many of the above-mentioned studies confirmed that SWC incidence was overall higher in BIMA, but that skeletonisation allows to significantly attenuate this increase [17, 20–22, 27]. Furthermore, the use of OPCAB might be associated with an additional reduction in SWC, as a post hoc analysis of the ART trial data showed that patients receiving BIMA grafts tended to have lower rates of SWC if they underwent OPCAB compared to on-pump CABG [27]. Another reason might be the perceived more complex procedures characteristic for OPCAB. However, we would like to mention that once the procedure is mastered and optimised, BIMA use in OPCAB allows for so-called ‘no aortic touch’ surgery, in which negligible trauma is done to the aorta. This could have the additional advantage of a reduction of cerebral microemboli and consequently less neurological complications, which are otherwise typical for CABG with cardiopulmonary bypass. Combined with the protective effect of OPCAB and skeletonisation on SWC, this could contribute to a reduction of morbidity and mortality.

### Limitations

This study has some limitations. First of all, this was a retrospective observational study and might therefore be susceptible to confounding. However, we identified patient characteristics and used multivariable models to account for characteristics that were different between groups. For example, the skeletonised group contained a lower proportion of BIMA and a greater prevalence of diabetic patients, which were both accounted for. Nevertheless, this does not exclude that there may have been unidentified confounders that were different between the two groups. Secondly, our study uses a before-and-after design, which might introduce an era effect. We can assure that perioperative management and the policy for wound prevention has remained largely the same for the period covered, and that only the incision drapes have become iodinated over the years. These might have been of minor influence, as a Cochrane review of two studies involving 1113 patients showed no effect of iodine-impregnated adhesive drapes on surgical site infection rate when compared to no drapes [36]. We could not adjust for changes in surgical, anesthesiologic and nursing staff however.

## Conclusions

In conclusion, this study shows in a large group of patients with a high proportion of BIMA grafts that, in combination with optimized wound management, skeletonised harvesting has a protective effect on the incidence and grade of SWC after OPCAB and is comparable to the conventional non-skeletonised technique regarding overall survival in patients with diabetes receiving BIMA grafts. It is suggested that the skeletonisation technique allows for safe application of BIMA grafting in these patients. This might help realise anaortic surgery for coronary revascularisation. Future studies might unveil more selective indications in which the use of skeletonisation is advised.

## Additional file

Additional file 1: **Table S1.** Multivariable model corresponding to Table 3 for SWC incidence. **Table S2.** Multivariable model corresponding to Table 3 for SWC grade. **Table S3.** Multivariable model corresponding to Table 3 for overall survival. **Table S4.** Multivariable model corresponding to Table 5 for SWC incidence. **Table S5.** Multivariable model corresponding to Table 5 for SWC grade. **Table S6.** Multivariable model corresponding to Table 5 for overall survival. **Table S7.** Multivariable model corresponding to Table 3 for SWC incidence (grade 1 excluded). **Table S8.** Multivariable model corresponding to Table 3 for SWC grade (grade 1 excluded). **Table S9.** Multivariable model corresponding to Table 5 for SWC incidence (grade 1 excluded). **Table S10.** Multivariable model corresponding to Table 5 for SWC grade (grade 1 excluded). (DOCX 23 kb)

## Data Availability

The datasets used and analysed during the current study are available from the corresponding author on reasonable request.

## Abbreviations

BIMA: Bilateral internal mammary artery
BMI: Body mass index
CABG: Coronary artery bypass graft
COPD: Chronic obstructive pulmonary disease
DM: Diabetes mellitus
eGFR: estimated glomerular filtration rate
IMA: Internal mammary artery
OPCAB: off-pump coronary artery bypass
RI: Renal insufficiency
SIMA: Single internal mammary artery
SWC: Sternal wound complication
TX: Transplantation
