# Supplementary material for: Skeletonisation contributing to a reduction of sternal wound complications: a retrospective study in OPCAB patients
Source: J Cardiothorac Surg. 2019 Sep 9;14:162. doi: 10.1186/s13019-019-0985-9 (PMC6734214; doi:10.1186/s13019-019-0985-9)
Supplement: Supplementary file 1 — Table S1. Multivariable model corresponding to Table 3 for SWC incidence. Table S2. Multivariable model corresponding to Table 3 for SWC grade. Table S3. Multivariable model corresponding to Table 3 for overall survival. Table S4. Multivariable model corresponding to Table 5 for SWC incidence. Table S5. Multivariable model corresponding to Table 5 for SWC grade. Table S6. Multivariable model corresponding to Table 5 for overall survival. Table S7. Multivariable model corresponding to Table 3 for SWC incidence (grade 1 excluded). Table S8. Multivariable model corresponding to Table 3 for SWC grade (grade 1 excluded). Table S9. Multivariable model corresponding to Table 5 for SWC incidence (grade 1 excluded). Table S10. Multivariable model corresponding to Table 5 for SWC grade (grade 1 excluded). (DOCX 23 kb) [file 13019_2019_985_MOESM1_ESM.docx]

**Table A1. Multivariable model corresponding to Table 3 for SWC incidence**

| **Model** | **Variable** | **Odds ratio (95% CI)** | ***P*** |
| --- | --- | --- | --- |
| **Uncorrected** | Group (skeletonised vs non-skeletonised) | 0.60 (0.37;0.98) | 0.041 |
| **Corrected^a^** | Group (skeletonised vs non-skeletonised) | 0.64 (0.38;1.04) | 0.072 |
|  | Diabetes mellitus (0 vs 3) | 0.25 (0.15;0.39) | <.001 |
|  | Diabetes mellitus (1 vs 3) | 0.87 (0.37;2.08) | 0.083 |
|  | Diabetes mellitus (2 vs 3) | 0.33 (0.18;0.59) | 0.023 |
|  | BIMA vs SIMA | 2.26 (1.49;3.41) | 0.001 |
| BIMA: bilateral internal mammary artery; SIMA: single internal mammary artery; SWC: sternal wound complication; CI: confidence interval.  ^a^ Corrected for diabetes mellitus and BIMA  P<0.05 was considered significant. | | | |

**Table A2. Multivariable model corresponding to Table 3 for SWC grade**

| **Model** | **Variable** | **Odds ratio (95% CI)** | ***P*** |
| --- | --- | --- | --- |
| **Uncorrected** | Group (skeletonised vs non-skeletonised) | 0.59 (0.36;0.97) | 0.036 |
| **Corrected^a^** | Group (skeletonised vs non-skeletonised) | 0.62 (0.38;1.02) | 0.062 |
|  | Diabetes mellitus (0 vs 3) | 0.24 (0.15;0.38) | <.001 |
|  | Diabetes mellitus (1 vs 3) | 0.91 (0.39;2.12) | 0.053 |
|  | Diabetes mellitus (2 vs 3) | 0.31 (0.17;0.56) | 0.014 |
|  | BIMA vs SIMA | 2.29 (1.51;3.46) | <.001 |
| BIMA: bilateral internal mammary artery; SIMA: single internal mammary artery; SWC: sternal wound complication; CI: confidence interval.  ^a^ Corrected for diabetes mellitus and BIMA  P<0.05 was considered significant. | | | |

**Table A3. Multivariable model corresponding to Table 3 for overall survival**

| **Model** | **Variable** | **Odds ratio (95% CI)** | ***P*** |
| --- | --- | --- | --- |
| **Uncorrected** | Group (skeletonised vs non-skeletonised) | 1.00 (0.52;1.91) | 1.000 |
| **Corrected^a^** | Group (skeletonised vs non-skeletonised) | 0.92 (0.48;1.75) | 0.791 |
|  | Diabetes mellitus (0 vs 1) | 0.69 (0.30;1.57) | 0.377 |
|  | Diabetes mellitus (0 vs 2) | 0.68 (0.47;0.97) | 0.031 |
|  | Diabetes mellitus (0 vs 3) | 0.85 (0.54;1.32) | 0.461 |
|  | Diabetes mellitus (1 vs 2) | 0.98 (0.42;2.31) | 0.963 |
|  | Diabetes mellitus (1 vs 3) | 1.23 (0.50;3.01) | 0.656 |
|  | Diabetes mellitus (2 vs 3) | 1.25 (0.75;2.08) | 0.385 |
|  | BIMA vs SIMA | 0.61 (0.46;0.82) | 0.001 |
| BIMA: bilateral internal mammary artery; SIMA: single internal mammary artery; SWC: sternal wound complication; CI: confidence interval.  ^a^ Corrected for diabetes mellitus and BIMA  P<0.05 was considered significant. | | | |

**Table A4. Multivariable model corresponding to Table 5 for SWC incidence**

| **Model** | **Variable** | **Odds ratio (95% CI)** | ***P*** |
| --- | --- | --- | --- |
| **Uncorrected** | Group (skeletonised vs non-skeletonised) | 0.41 (0.19;0.88) | 0.023 |
| **Corrected^a^** | Group (skeletonised vs non-skeletonised) | 0.47 (0.22;1.03) | 0.058 |
|  | BIMA vs SIMA | 2.04 (1.13;3.70) | 0.019 |
| BIMA: bilateral internal mammary artery; SIMA: single internal mammary artery; SWC: sternal wound complication; CI: confidence interval.  ^a^ Corrected for diabetes mellitus and BIMA  P<0.05 was considered significant. | | | |

**Table A5. Multivariable model corresponding to Table 5 for SWC grade**

| **Model** | **Variable** | **Odds ratio (95% CI)** | ***P*** |
| --- | --- | --- | --- |
| **Uncorrected** | Group (skeletonised vs non-skeletonised) | 0.41 (0.19;0.88) | 0.022 |
| **Corrected^a^** | Group (skeletonised vs non-skeletonised) | 0.47 (0.21;1.02) | 0.055 |
|  | BIMA vs SIMA | 2.07 (1.14;3.75) | 0.017 |
| BIMA: bilateral internal mammary artery; SIMA: single internal mammary artery; SWC: sternal wound complication; CI: confidence interval.  ^a^ Corrected for diabetes mellitus and BIMA  P<0.05 was considered significant. | | | |

**Table A6. Multivariable model corresponding to Table 5 for overall survival**

| **Model** | **Variable** | **Odds ratio (95% CI)** | ***P*** |
| --- | --- | --- | --- |
| **Uncorrected** | Group (skeletonised vs non-skeletonised) | 0.74 (0.21;2.54) | 0.630 |
| **Corrected^a^** | Group (skeletonised vs non-skeletonised) | 0.68 (0.20;2.34) | 0.535 |
|  | BIMA vs SIMA | 0.71 (0.44;1.14) | 0.151 |
| BIMA: bilateral internal mammary artery; SIMA: single internal mammary artery; SWC: sternal wound complication; CI: confidence interval.  ^a^ Corrected for diabetes mellitus and BIMA  P<0.05 was considered significant. | | | |

**Table A7. Multivariable model corresponding to Table 3 for SWC incidence (grade 1 excluded)**

| **Model** | **Variable** | **Odds ratio (95% CI)** | ***P*** |
| --- | --- | --- | --- |
| **Uncorrected** | Group (skeletonised vs non-skeletonised) | 0.71 (0.50;1.02) | 0.067 |
| **Corrected^a^** | Group (skeletonised vs non-skeletonised) | 0.74 (0.51;1.07) | 0.114 |
|  | Diabetes mellitus (0 vs 3) | 0.29 (0.20;0.43) | <.001 |
|  | Diabetes mellitus (1 vs 3) | 0.55 (0.24;1.28) | 0.782 |
|  | Diabetes mellitus (2 vs 3) | 0.41 (0.25;0.66) | 0.202 |
|  | BIMA vs SIMA | 2.29 (1.65;3.16) | <.001 |
| BIMA: bilateral internal mammary artery; SIMA: single internal mammary artery; SWC: sternal wound complication; CI: confidence interval.  ^a^ Corrected for diabetes mellitus and BIMA  P<0.05 was considered significant. | | | |

**Table A8. Multivariable model corresponding to Table 3 for SWC grade (grade 1 excluded)**

| **Model** | **Variable** | **Odds ratio (95% CI)** | ***P*** |
| --- | --- | --- | --- |
| **Uncorrected** | Group (skeletonised vs non-skeletonised) | 0.70 (0.49;1.01) | 0.054 |
| **Corrected^a^** | Group (skeletonised vs non-skeletonised) | 0.727 (0.502;1.05) | 0.091 |
|  | Diabetes mellitus (0 vs 3) | 0.28 (0.19;0.41) | <.001 |
|  | Diabetes mellitus (1 vs 3) | 0.60 (0.27;1.36) | 0.541 |
|  | Diabetes mellitus (2 vs 3) | 0.39 (0.24;0.62) | 0.105 |
|  | BIMA vs SIMA | 2.30 (1.67;3.18) | <.001 |
| BIMA: bilateral internal mammary artery; SIMA: single internal mammary artery; SWC: sternal wound complication; CI: confidence interval.  ^a^ Corrected for diabetes mellitus and BIMA  P<0.05 was considered significant. | | | |

**Table A9. Multivariable model corresponding to Table 5 for SWC incidence (grade 1 excluded)**

| **Model** | **Variable** | **Odds ratio (95% CI)** | ***P*** |
| --- | --- | --- | --- |
| **Uncorrected** | Group (skeletonised vs non-skeletonised) | 0.38 (0.20;0.71) | 0.003 |
| **Corrected^a^** | Group (skeletonised vs non-skeletonised) | 0.44 (0.23;0.83) | 0.012 |
|  | BIMA vs SIMA | 2.17 (1.32;3.55) | 0.002 |
| BIMA: bilateral internal mammary artery; SIMA: single internal mammary artery; SWC: sternal wound complication; CI: confidence interval.  ^a^ Corrected for diabetes mellitus and BIMA  P<0.05 was considered significant. | | | |

**Table A10. Multivariable model corresponding to Table 5 for SWC grade (grade 1 excluded)**

| **Model** | **Variable** | **Odds ratio (95% CI)** | ***P*** |
| --- | --- | --- | --- |
| **Uncorrected** | Group (skeletonised vs non-skeletonised) | 0.38 (0.20;0.71) | 0.003 |
| **Corrected^a^** | Group (skeletonised vs non-skeletonised) | 0.43 (0.23;0.83) | 0.011 |
|  | BIMA vs SIMA | 2.18 (1.33;3.57) | 0.002 |
| BIMA: bilateral internal mammary artery; SIMA: single internal mammary artery; SWC: sternal wound complication; CI: confidence interval.  ^a^ Corrected for diabetes mellitus and BIMA  P<0.05 was considered significant. | | | |
